# CircRNA Regulation of T Cells in Cancer: Unraveling Potential Targets

**DOI:** 10.3390/ijms25126383

**Published:** 2024-06-09

**Authors:** Zelin Li, Shuanshuan Yin, Kangping Yang, Baojie Zhang, Xuanhuang Wu, Meng Zhang, Dian Gao

**Affiliations:** 1 School of Basic Medical Sciences, Jiangxi Medical College, Nanchang University, Nanchang 330047, China; 4203121208@email.ncu.edu.cn (Z.L.); 406400230084@email.ncu.edu.cn (B.Z.); 2 The First Clinical Medical College, Nanchang University, Nanchang 330047, China; 4202121028@email.ncu.edu.cn (S.Y.); 4204122017@email.ncu.edu.cn (X.W.); 3 The Second Clinical Medical College, Nanchang University, Nanchang 330047, China; yangkangping0913@email.ncu.edu.cn

**Keywords:** circular RNAs, T cells, cancer, immune checkpoint inhibitors, tumor microenvironment

## Abstract

T lymphocytes play a critical role in antitumor immunity, but their exhaustion poses a significant challenge for immune evasion by malignant cells. Circular RNAs (circRNAs), characterized by their covalently closed looped structure, have emerged as pivotal regulators within the neoplastic landscape. Recent studies have highlighted their multifaceted roles in cellular processes, including gene expression modulation and protein function regulation, which are often disrupted in cancer. In this review, we systematically explore the intricate interplay between circRNAs and T cell modulation within the tumor microenvironment. By dissecting the regulatory mechanisms through which circRNAs impact T cell exhaustion, we aim to uncover pathways crucial for immune evasion and T cell dysfunction. These insights can inform innovative immunotherapeutic strategies targeting circRNA-mediated molecular pathways. Additionally, we discuss the translational potential of circRNAs as biomarkers for therapeutic response prediction and as intervention targets. Our comprehensive analysis aims to enhance the understanding of immune evasion dynamics in the tumor microenvironment by facilitating the development of precision immunotherapy.

## 1. Introduction

The presence of immune cell infiltrates within tumors is a recognized prognostic marker for various cancers; however, the native antitumor immune response is frequently insufficient to arrest disease progression [1]. Within the tumor microenvironment (TME), immune checkpoint proteins such as programmed death-ligand 1 (PD-L1) and its homolog PD-L2 play crucial roles as modulators of immune responses [2]. PD-L1 is recognized for its ability to suppress T cell cytotoxicity against tumor cells, while PD-L2 has been shown to markedly attenuate T cell receptor (TCR)-driven proliferation and cytokine production in CD4^+^ T cells, indicating overlapping yet distinct immunosuppressive functions [3]. Interventions targeting the PD-L1/PD-1 axis reinvigorate T cell effector functions [4,5]. Nonetheless, the majority of patients exhibit only partial responses to PD-L1/PD-1 axis inhibitors, a phenomenon potentially ascribed to the interplay between immune checkpoint inhibition and pervasive immunosuppression within the tumor microenvironment, culminating in suboptimal T cell activation [5]. The influence of the metabolic states of other immune cell types, such as natural killer (NK) cells, on the efficacy of immunotherapy is a relatively uncharted research frontier [6].

CD8^+^ cytotoxic T lymphocytes (CTLs) are instrumental in the direct elimination of cancer cells due to their ability to enhance the tumor microenvironment through the deployment of cytotoxic granules, the expression of Fas ligands, and the production of some cytokines such as tumor necrosis factor-alpha (TNF-α) and interferon-gamma (IFN-γ), highlighting their critical role in antitumor immunity [7]. Additionally, CD4^+^ T cells augment the efficacy of antigen-presenting cells (APCs) and stimulate the proliferation of CD8^+^ T cells through the secretion of interleukin-2 (IL-2), thus amplifying their cytotoxic potential and reinforcing antitumor immune responses within the tumor microenvironment [7]. The enhancement of T lymphocyte immune surveillance and activation, coupled with the reduction in immune tolerance, is a promising avenue for strengthening anticancer immunity. T cells are acknowledged as central to the success of cancer immunotherapy, with the depletion of T cells being a significant factor that limits the efficacy of such treatments [8,9]. Consequently, efforts to restore antitumor T cell function focus on understanding T cell metabolic reprogramming, regulation by transcription factors, and modulation of immune checkpoint pathways [10,11,12].

Emerging evidence has implicated circRNAs as significant modulators of immune cell function and disease progression, including critical aspects of T cell activation, proliferation, and immune response regulation [13,14]. Despite these insights, the intricate mechanisms by which circRNAs participate in tumor immunotherapy have not yet been fully delineated. A profound understanding of the mechanisms and functions of T cells in tumorigenesis, alongside the regulatory influence of circRNAs, is essential for advancing our grasp of the molecular etiology of T cell dysfunction. Such knowledge forms the theoretical foundation for developing innovative immunotherapeutic strategies [15,16]. This manuscript aims to provide an in-depth exploration of the mechanisms by which circRNAs modulate T lymphocytes within the context of tumors and to discuss their prospective clinical applications.

## 2. T Cells in Cancer

Some T cells differentiate into regulatory T cells (Tregs), which express the immune checkpoint receptor cytotoxic T lymphocyte-associated antigen-4 (CTLA-4), inhibiting the immune response of tumor-specific T cells and aiding tumors in evading immune surveillance [17]. Notably, certain circular RNAs (circRNAs) have been shown to enhance Treg-cell-mediated immune suppression, particularly during anti-CTLA-4 therapy, suggesting that circRNAs modulate the tumor immune landscape [18,19].

### 2.1. Changes in the Function and Status of T Cells in TME

Although tumor-associated antigen (TAA)-specific T cells exist, their functional impairment within the tumor microenvironment often hinders their antitumor effects, allowing tumors to persist [20]. T cell failure manifests through the upregulation of inhibitory receptors such as PD-1 and CTLA-4, impaired effector function, and metabolic dysregulation, preventing effective tumor control. In addition to PD-1 and CTLA-4, B7-H3 is a crucial immune checkpoint molecule that plays a role in the tumor microenvironment. B7-H3 is highly expressed in a range of tumors and is considered to be one of the mechanisms promoting tumor immune escape [21]. B7-H3 can inhibit the activation and proliferation of T cells and hinder the antitumor immune response mediated by T cells. Therefore, B7-H3 expression may lead to T cell dysfunction and tumor immune escape, hindering the efficacy of T-cell-based immunotherapies [22].

T cells play an important role in the immune response against tumors, and CD8+ cytotoxic T cells (CTLSs) eliminate tumor cells by recognizing and binding to tumor-associated antigens on the surface of tumor cells and releasing cytotoxic particles. CTLs can also secrete inflammatory factors IFN-γ and TNF-α to enhance the antitumor immune microenvironment. CD4+ T helper cells secrete cytokine IL-2 to stimulate the proliferation of CTLs and other immune cells, promote the maturation of antigen-presenting cells (APCs), and enhance the ability of CTLs to recognize and kill tumor cells. However, the tumor microenvironment fosters an immunosuppressive milieu, compromising T cell function. Multiple mechanisms are involved in the immunosuppression of the TME, including immune checkpoint molecules such as PD-1, CTLA-4, and B7-H3, along with immunosuppressive cells. Regulatory T cells (Tregs) are an important subset of T cells and are also a group of immunosuppressive cells. These cells express the transcription factor FoxP3 and inhibit the immune response. On the one hand, high levels of Tregs in the TME can suppress antitumor immunity by secreting the inhibitory cytokines IL-10 and TGF-β or by suppressing the activity of effector T cells, including chimeric antigen receptor (CAR)-T cells and T cell receptor (TCR)-T cells, through a cell-contact-dependent mechanism. On the other hand, moderate Tregs also help to maintain immune balance and prevent autoimmune damage. In general, an increased proportion of Tregs in the tumor microenvironment is often associated with poor prognosis [23]. The presence of these different T cell subsets and their interactions impede the ability of tumor-infiltrating T cell therapies, such as CAR-T cell therapy, to efficiently penetrate and kill tumor cells in solid tumors. Therefore, exploring the molecular regulatory mechanisms that affect the dynamic balance and functional status of T cell subsets is crucial for improving the efficacy of tumor immunotherapy. Recent findings indicate that circRNAs may regulate T cell responses in cancer [24], with the dysregulation of circRNAs associated with T cell dysfunction and resistance to PD-1 therapy [25,26]. Elucidating the mechanisms by which circRNAs modulate T cells could unveil new therapeutic targets to restore antitumor immunity.

Therefore, elucidating how circRNAs influence T cell functional states within the tumor microenvironment holds promise for developing more effective cancer immunotherapies capable of overcoming current limitations [14].

### 2.2. Immunotherapy Targeting T Cells

Numerous T-cell-based immunotherapy approaches have emerged for cancer treatment, aiming to bolster antitumor immune responses and enhance tumor immunogenicity [27,28]. These strategies include immune checkpoint blockade, the reinforcement of costimulatory signals, CAR-T cells, TCR-T cells, and tumor-associated antigen vaccines [29]. A summary of these strategies is presented in Table 1.

Among these strategies, CAR-T cell and TCR-T cell immunotherapies have garnered significant clinical attention in recent years. CAR-T cells are generated from patient-derived T cells engineered to express chimeric antigen receptors targeting tumor-associated antigens such as EpCAM, giving them the ability to selectively identify and eliminate tumor cells [36]. Conversely, TCR-T cells involve the substitution of TCR genes in T cells with specific TCRs that recognize tumor-associated antigens [37]. While both therapies have demonstrated considerable clinical efficacy, they face challenges such as T cell exhaustion.

Immune checkpoint molecules such as CTLA-4 and PD-1 impede T cell function upon binding to their ligands, causing T cells to lose their tumor-killing ability. Consequently, inhibiting these immunosuppressive pathways is a pivotal strategy for augmenting CAR-T/TCR-T cell efficacy. Recent investigations into circRNA, an emerging noncoding RNA molecule, have revealed its capacity to modulate the expression of tumor-associated antigens such as EpCAM [38,39], suggesting a potential impact on CAR-T cell recognition proficiency. Furthermore, circRNAs have been implicated in regulating the expression of immune checkpoint molecules and participating in immune modulation [18,40].

Given these findings, we propose that integrating circRNA regulatory strategies into CAR-T/TCR-T cell immunotherapy represents a promising frontier in cancer immunotherapy. On the one hand, optimizing the targeting capability of CAR-T cells can be achieved by modulating the expression of antigen-associated circRNAs, such as EpCAM. On the other hand, the modulation of circRNA expression linked to immunosuppressive pathways, or the use of exogenous circRNA interventions, holds promise for enhancing T cell antitumor activity and prolonging T cell persistence, thereby bolstering T cell efficacy. Hence, circRNAs have emerged as pivotal regulatory elements in integrated CAR-T/TCR-T cell immunotherapy.

In summary, existing therapeutic strategies have both advantages and disadvantages [41].

## 3. Structure and Function of circRNAs

Circular RNAs (circRNAs) constitute a class of circular noncoding RNAs generated through nonlinear back-splicing events. They are categorized into three main categories: exonic circRNAs (EcRNAs), intronic circRNAs (CiRNAs), and exon-intronic circRNAs (EicRNAs) [42,43]. There is also an important class called extrachromosomal circular DNAs (eccDNA). EccDNAs are DNA molecules that exist independently of chromosomes and have circular structures. As a new type of extrachromosomal genetic element, eccDNA plays an important role in multiple processes, such as tumorigenesis, development, heterogeneity, and drug resistance [44,45]. Similarly, eccDNA can also be used as a template for circular RNA production. CircRNAs are abundant in humans and other mammals and are highly conserved across species. In recent years, circRNAs have been found to play important regulatory roles in cellular processes, particularly in the occurrence and development of cancer.

Initially regarded as aberrant splicing byproducts, circRNAs are now recognized as abundant molecules found across diverse organisms. They form complex regulatory networks by interacting with microRNAs (miRNAs) and other RNA-binding proteins (RBPs) [46].

Many circRNA sequences contain miRNA recognition elements and function as “sponges” for miRNAs. By sequestering miRNAs, circRNAs can enhance or inhibit their regulatory effects on target genes. This absorption of miRNAs by circRNAs modulates gene expression by altering the regulatory impact of miRNAs on their target mRNAs [47,48]. For instance, ciRS-7 harbors numerous highly conserved miR-7 binding sites, effectively sequestering miR-7 and suppressing its activity, thus leading to the upregulation of miR-7 target genes [49,50]. Additionally, some circRNAs regulate the expression of tumor-related genes and immune checkpoint molecules through this molecular mechanism. For example, circRNA_104889 can modulate the miR-34a/miR-28-5p/PD-L1/PD-1 axis, affecting the expression levels of PD-L1 and PD-1 in lung adenocarcinoma, thus regulating T cell activity [51].

**Figure 1:**
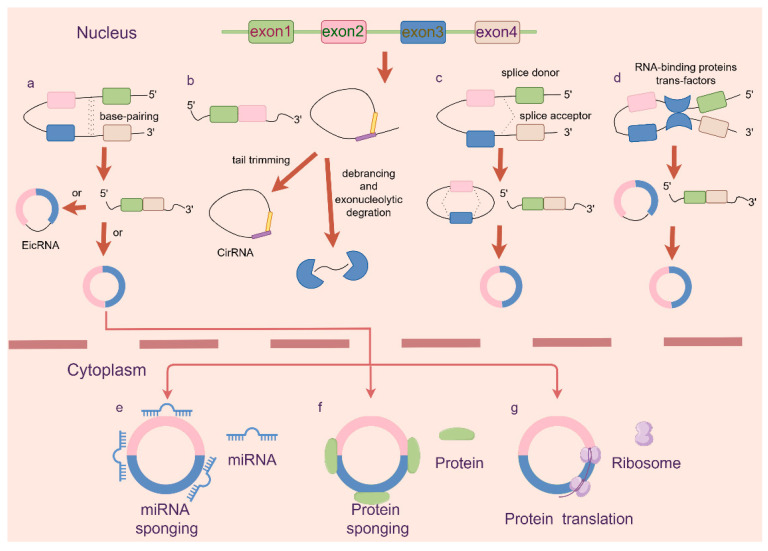
Formation and functional mechanisms of circRNAs. CircRNAs, arising from diverse splicing events, play multifaceted roles in cellular processes. The schematic representation illustrates their formation and key functional mechanisms. (**a**) Back-splicing. Circularization occurs through nonlinear back-splicing, resulting in stable circRNAs. (**b**) Intron pairing. Intronic regions contribute to circRNA formation, potentially influencing gene regulation. (**c**) Exon skipping. Exonic circRNAs (EcRNAs) emerge from skipped exons, adding to the circRNA repertoire. (**d**) RNA-binding protein-mediated splicing. RNA-binding proteins facilitate circRNA biogenesis. The pivotal roles of circRNAs include (**e**) miRNA sponges. circRNAs sequester miRNAs, modulating gene expression networks. (**f**) Protein interaction. CircRNAs interact with proteins to form regulatory complexes. (**g**) Micropeptide encoding. Some circRNAs encode micropeptides that potentially influence cellular functions. CircRNAs impact gene expression at the transcriptional and translational levels. (Figure created using Figdraw.)

CircRNA interactions with RBPs can influence the stability, splicing, transport, and translation of target mRNAs, modulating protein expression levels [52]. For example, circTHBS1 drives gastric cancer progression by increasing INHBA mRNA expression and stability in an RBP-dependent manner [53] (Figure 1).

Furthermore, some circRNAs undergo translation via internal ribosome entry sites (IRESs), producing micropeptides that influence translation internally. However, further research is needed to understand how this function affects the development and progression of tumors.

## 4. CircRNA Regulation of T Cells in Cancer

**Figure 2:**
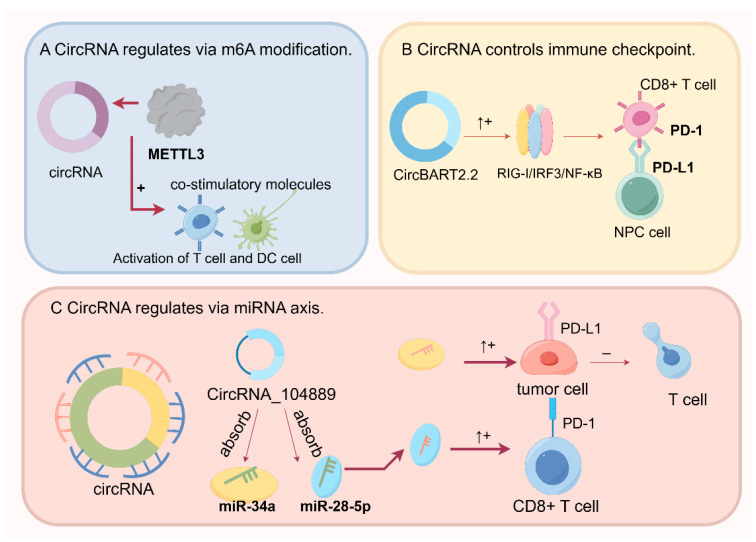
Modulation of the tumor immune response by circular RNAs (circRNAs) via m6A methylation, miRNA sponging, and the regulation of immune checkpoints. (**A**) Effect of METTL3 suppression on dendritic and T cell maturation: diminished METTL3 expression within dendritic cells (DCs) and T cells impairs their maturation, as evidenced by a reduction in the expression of costimulatory molecules. This maturation deficit subsequently compromises the capacity of DCs to effectively activate T cells in vitro. (**B**) The role of circBART2.2 in enhancing the expression of PD-L1 in nasopharyngeal carcinoma (NPC): PD-L1 expression is increased in NPC tissues with elevated levels of circBART2.2 via the RIG-I/IRF3/NF-κB signaling cascade. As a result of increased PD-L1 expression within the tumor microenvironment, T cells are induced to apoptose. (**C**) Regulation of PD-1 and PD-L1 expression by circRNA_104889 in lung adenocarcinoma (LUAD): CircRNA_104889 expression in LUAD modulates miR-34a/miR-28-5p function to modulate PD-1 and PD-L1. This interaction has downstream effects on T cell activity and potentially influences the tumor immune landscape. (Figure created using Figdraw.)

The burgeoning field of circRNA research has unveiled two pivotal inquiries concerning their involvement in oncogenesis: What are the regulatory mechanisms governing circRNA expression during cancer development and progression? How do circRNAs contribute to the hallmarks of cancer, including therapy resistance, and their potential as biomarkers? This section examines how circRNAs interact with T cell biology in the tumor microenvironment. Emerging evidence suggests that the TME exhibits dysregulated circRNA expression, modulating microRNA (miRNA) availability through sponge-like mechanisms. Moreover, circRNAs packaged within extracellular vesicles, such as exosomes, profoundly influence T cell proliferation, activation, and tumor cell metastasis. These circRNAs may play a role in fostering antitumor immune resistance, either as tumor promoters or suppressors, by facilitating intercellular communication between tumors and immune cells [54]. CircRNAs activate dendritic cells to boost antigenic cross-reactivity and CD4+ and CD8+ T cells [55]. It is also possible that circRNAs can directly affect immune cells, including T cells. However, it remains unclear whether and how circRNAs control CD4+ and CD8+ T cells. Despite these insights, the precise role of circRNAs in modulating T cell immune responses within the TME requires further elucidation. It is hypothesized that changes in T cell secretory mediators within the TME may correlate with alterations in circRNA expression levels and the miRNA regulatory network, potentially fostering tumorigenesis [56]. CircRNAs can influence tumor immunity through interactions with signaling pathways. CircRNAs modulate N6-methyladenosine (m6A) modification, a pivotal epigenetic event influencing tumor progression and the interplay between miRNAs and immune checkpoints. Initially underestimated, the functional significance of circRNAs in cellular processes, particularly in cancer progression, is garnering increasing recognition. Understanding the intricate regulatory networks involving circRNAs is imperative for deciphering the molecular mechanisms underlying T cell dysfunction and resistance in cancer, thereby offering avenues for the development of novel therapeutic strategies (Figure 2).

### 4.1. CircRNA Regulation by N6-Methyladenosine (m6A) Modification in Cancer

Recent advances have elucidated the intricate relationship between circRNAs and m6A modifications, a form of RNA epigenetic alteration, in cancer biology [57,58,59]. CircRNAs have emerged as substrates for m6A methylation, and intriguingly, they can reciprocally modulate the m6A landscape by competitively binding to m6A regulatory proteins or directly interacting with them [58,59]. This reciprocal regulatory mechanism underscores the complexity of post-transcriptional gene expression control. m6A modification significantly influences the translational efficiency and decay of circRNAs, thereby impacting tumorigenesis and cancer progression [60]. CircRNAs are proposed to be shielded from immune detection by m6A modifications, potentially evading innate immune surveillance through this molecular signature [61]. ALKBH5-mediated m6A modification of circCCDC134, for example, enhances HIF1A transcription, facilitating cervical cancer metastasis [62]. Prostate cancer progression is facilitated by PDHA1-mediated mitochondrial respiration regulation through m6A-modified circRBM33 [62]. This suggests that m6A-modified circRNAs could play a crucial role in various cancers by modulating tumor growth, invasion, and progression, with implications for immune regulation, T cell activation, and immune surveillance [63,64,65,66]. METTL3, part of the m6A methyltransferase complex, enhances the expression of immune costimulatory molecules, thereby stimulating T cell activation [64]. Conversely, the downregulation of METTL3 in DCs can impair DC maturation and the expression of costimulatory molecules, leading to reduced T cell activation in vitro [64]. M6A modification is pivotal to immune cell function, as demonstrated by these findings (Figure 2A).

Additionally, m6A modification of circRNAs is associated with cyclin-dependent kinase 1 (CDK1) upregulation, which regulates T cell proliferation [67]. This finding underscores the multifaceted role of m6A in not only governing circRNA expression, but also directly influencing the immune response within the tumor microenvironment. Despite these insights, the precise mechanisms governing the interaction between m6A modifications and circRNAs remain incompletely understood. Future research efforts should focus on identifying specific targets of m6A-modified circRNAs and understanding how their expression can be regulated to impact tumor immunity.

### 4.2. Regulation of the miRNA Axis by CircRNAs in Cancer Progression

Circular RNAs (circRNAs) exert significant regulatory effects on cancer progression by acting as miRNA sponges, protein templates, and scaffolds [68]. For instance, ciRS-7 facilitates proliferation by sequestering miR-7 and subsequently upregulating EGFR and RAF1 expression [69,70]. CircRNAs serve as miRNA sponges, modulating the expression of miRNA target genes in recipient cells, thereby influencing downstream signaling molecules and pathways associated with tumor immunity and ultimately impacting T cell function in tumor immunity. Through the upregulation of circRNA-SORE, which sequesters miR-103a-2-5p and miR-660-3p via miRNA sponges, sorafenib resistance is induced in tumor cells [69]. Additionally, elevated expression of circRNA_104889 in lung adenocarcinoma influences PD-1 and PD-L1 expression via the miR-34a/miR-28-5p/PD-L1/PD-1 axis, thereby affecting T cell activity [51]. In hepatocellular carcinoma tumorigenesis, the circRNA CCND1 expedites cell metastasis and proliferation by HMGA2/miR-497-5p modulation [71]. CircRNAs bind to miRNAs in a manner that allows endogenous RNAs to compete for mRNAs corresponding to downstream targets of miRNAs, increasing the likelihood of the progression of breast cancer [62]. For downregulated circRNAs, the restoration of tumor suppressor gene circRNA levels can be achieved through cloning the circRNA sequence and its regulatory flanking region or through the overexpression of circRNA via artificial synthesis based on their specific circRNA–miRNA axis [58,72]. Given the regulatory importance of the sponge mechanism, the circRNA–miRNA network plays a crucial role in cancer regulation, where the aberrant expression of upstream circRNAs can impact downstream miRNA and mRNA expression. Therefore, efficient and precise modulation during tumorigenesis can be achieved through a deeper understanding of the circRNA–miRNA interaction axis.

### 4.3. CircRNAs Regulate Immune Checkpoints

In order to evade immune surveillance, tumor cells upregulate immune checkpoint molecules such as PD-L1 and CTLA-4 [73]. These mechanisms include the programmed cell death pathway (PCD), an immune checkpoint mediated by circRNAs. Research indicates that alterations in circRNA abundance within tumor tissues can influence T cell function by modulating the expression of immune checkpoint proteins such as PD-1 and PD-L1. It has been shown that inhibiting circ-PTPN22 expression enhances T cell infiltration, elevates responses to anti-PD-L1 therapy, and augments CD8+ T cell activation, increasing susceptibility to checkpoint inhibitors [74]. A high level of circ-PTPN22 correlates directly with the enhanced expression of immune checkpoint genes and regulatory T cells across various cancers. Additionally, specific circRNAs derived from certain cancer types, such as ovarian-cancer-derived circ-0001068 and hepatocellular-carcinoma-derived circCCAR1, have been implicated in PD-1 regulation in T cells, leading to immune evasion and resistance to anti-PD1 therapy [55,75]. circBART2.2 overexpressed has been observed in nasopharyngeal carcinoma tissues, upregulating the expression of PD-L1. This decreases T cell infiltration and apoptosis by inhibiting T-cell-mediated cytotoxicity [76]. Leveraging circRNAs to modulate immune checkpoints is a promising approach for mitigating T cell depletion in antitumor immunity. Furthermore, evidence suggests that circRNAs may activate dendritic cells to promote antigenic cross-reactivity and activate CD4+ and CD8+ T cells, although the direct impact of circRNAs on various immune cell types warrants further investigation [77]. Exploring cancer-specific circRNAs, elucidating miRNA–circRNA and circRNA–RBP interactions, and predicting the variable splicing of related genes offer novel insights into cancer therapy. Additionally, circRNA signatures hold clinical significance in predicting cancer recurrence and progression, underscoring the importance of identifying potential therapeutic targets.

### 4.4. CircRNAs Trigger the Tumor Immune Response

Recent research has demonstrated that circRNAs can produce cryptic antigenic peptides via a nonclassical translation pathway, thereby leading to an immune response. Mice that were restimulated in vitro with dendritic cells transfected with circFam53b and tumor cells expressing circFam53b exhibited an increase in CD4+ and CD8+ T cells, as well as perforin production by cytotoxic T lymphocytes. This suggests a suppression of tumor growth, highlighting the immunogenic and antigen-specific properties of circFam53b, which is capable of triggering an antitumor immune response [78]. Lactate produced by KRASMUT tumor cells can facilitate CTL access through MCT1, causing histone lactylation and activating circATXN7 transcription. circATXN7 inhibits the expression of NF-κB-mediated genes, including genes that can inhibit T cell apoptosis. This process increases the likelihood of activation-induced cell death (AICD) in CTLs in KRASMUT tumors, decreasing the quantity and effectiveness of these immune cells and ultimately leading to tumor immune escape [79]. It has been discovered that tumor cells release exosomes which are temporarily internalized by activated CD8+ T cells, thereby suppressing cytotoxicity and cytokine secretion, as well as inhibiting CD8+ T cell proliferation [25]. This mechanism by which circRNAs influence T cell activity, and consequently the tumor immune response through exosomal transport, encoding, or binding of related substances, is innovative and significant, and the interactions involved can enhance tumor progression. Whether exogenous circRNAs can induce immune responses specific to antigens and serve as antigens for educating T cells remains unclear. Hence, it is crucial to explore the ways in which circRNAs impact T cells in various tumor microenvironments(Table 2).

## 5. Potential Clinical Applications

CircRNAs, which are predominantly present in exosomes, are emerging as key mediators of intercellular communication within the tumor microenvironment. Their role as stable templates for therapeutic protein delivery further highlights their multifaceted clinical potential. Given their stability and specificity, circRNAs are increasingly recognized for their utility in cancer diagnosis and early-stage screening. Research has highlighted the significant role of circRNAs in modulating immune cell function, thereby influencing cancer progression. In particular, they have been shown to affect the activation, proliferation, and exhaustion of T cells, which are critical for mounting an effective immune response against cancer. Understanding these processes could provide novel insights into T cell biology within the context of cancer and inform the development of targeted immunotherapeutic strategies. Additionally, circRNAs, including their sensitivity and resistance profiles, hold promise as predictive biomarkers for cancer recurrence, metastasis, and response to chemotherapy [93]. The identification of circRNA-based targets for immunotherapy and the discovery of biomarkers for tumor diagnosis and prognosis could significantly impact the clinical management of cancer. Although progress has been made in exploring circRNAs for diagnostic and therapeutic applications, the potential of circRNA-based cancer vaccines remains largely uncharted. Future research aimed at evaluating the feasibility and efficacy of circRNA vaccines in tumor immunotherapy could lead to innovative approaches that enhance vaccine stability and the immune response, offering a promising direction for advancing cancer treatment.

### 5.1. Tumor Immunotherapy Targets

**Figure 3:**
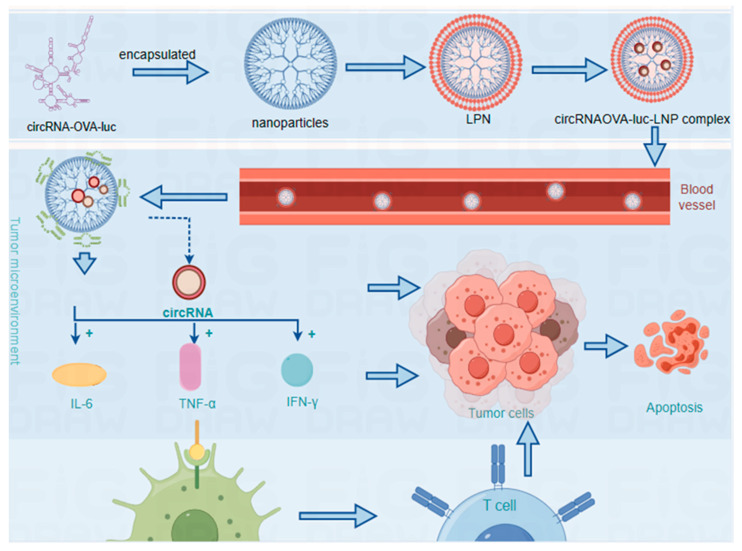
Circular RNAOVA-luc delivered by lipid nanoparticles activates antigen-specific T cells and inhibits tumor growth. CircumRNAOVA-luc-LNP vaccine complex promotes strong antigen-specific T lymphocyte responses and has antitumor properties. (Figure created using Figdraw.)

Therapeutic strategies based on RNA, particularly synthetic circRNAs, are gaining momentum in the realms of research and clinical development due to their potential to augment immunotherapy [94,95]. Exonucleases cannot degrade circRNAs because of their highly conserved sequences and circular structures. In addition to its relatively stable structure, it is highly expressed and participates in gene transcription and post-transcriptional regulation. Hence, circRNAs have a long half-life in the cell, allowing them to perform their functions more permanently than other noncoding RNAs. By modulating the expression of enclosed miRNAs, circRNAs can also regulate target gene expression. As miRNA sponges, they regulate miRNA activity and play a role in protein translation, exhibiting multiple functions and having natural advantages over other noncoding RNAs in cancer therapeutics. Exosomes, as natural vesicles for intercellular communication, have been recognized for their utility in targeted drug delivery to specific organs [96]. Within this context, circRNAs, which act as stable bioactive components of exosomes, have been leveraged to enhance anticancer immunity. They modulate the transcriptome of recipient cells and influence tumor-associated signaling pathways, thereby impacting the tumor microenvironment [97]. Recent advancements have highlighted the effectiveness of lipid nanoparticle (LNP) systems for the delivery of circRNAs. CircRNAOVA-luc-LNP vaccine complexes have demonstrated the capacity to induce robust antigen-specific T cell responses and potent antitumor effects [98] (Figure 3). The distinctive structural attributes of circRNAs, including their resistance to degradation and the ease of loading and targeting modifications, render them highly valuable for the development of immunotherapies with improved efficiency and reduced toxicity. Nonetheless, challenges such as the short half-life of nanoparticles and constraints on the size of encapsulated molecules necessitate ongoing technological innovations to ensure the efficacy of circRNA delivery.

### 5.2. Tumor Diagnostic and Prognostic Markers

**Figure 4:**
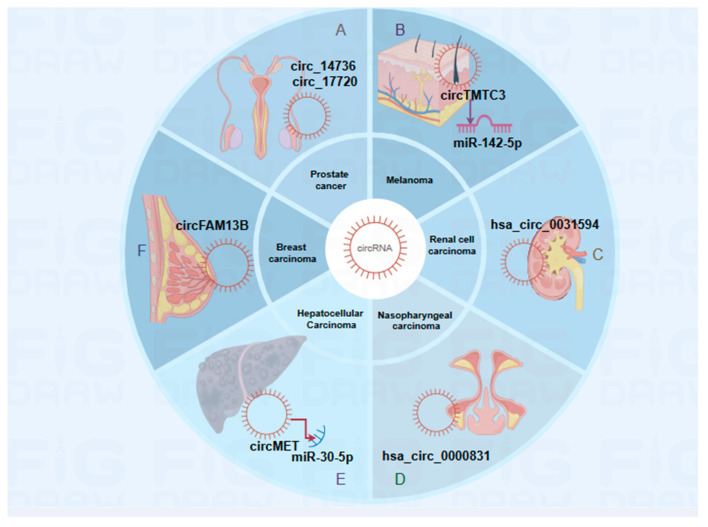
CircRNAs as diagnostic and prognostic markers in oncology. The figure illustrates the potential of circRNAs as markers for various cancers. (**A**–**F**) CircRNAs serve as diagnostic and prognostic biomarkers in different cancers by specific mechanism. (Figure created using Figdraw.)

Unlike other linear RNAs, circRNAs exhibit stable expression and are not affected by splicing heterogeneity or the cellular environment. Therefore, they can potentially serve as biomarkers. High-throughput sequencing studies have revealed the potential of exosomal circRNAs as diagnostic and prognostic biomarkers in oncology. Distinct circRNA expression profiles have been observed between cancerous and normal tissues, and bioinformatic analyses have facilitated their identification [99,100]. The circFAM13B gene, for instance, has been shown to suppress tumor immune evasion and enhance immunotherapy sensitivity in breast cancer patients [99]. In contrast, the aberrant expression of circPRDM4 in hepatocellular carcinoma has been linked to increased PD-L1 levels, facilitating immune evasion mediated by CD8+ T cells [100]. Despite their promise, further research is essential to confirm the clinical relevance of circRNAs as exosomal biomarkers for tumor diagnosis [99,100]. (Refer to Figure 4 and Table 3 for further details.)

### 5.3. Modulation of Tumor Metastasis and Invasion

There is a growing body of evidence implicating circRNA expression levels in the modulation of tumor metastasis and invasion. CircRNA-based therapeutics require pharmacokinetic studies to ensure their safety and efficacy [117]. They can act as miRNA sponges, neutralizing miRNAs and alleviating their suppression of target genes, thus promoting metastatic potential [118,119]. A number of signaling pathways are regulated by circRNAs, such as Wnt and PI3K/AKT pathways, which are key to cancer cell invasion and metastasis [118,119]. The interplay between the tumor microenvironment and circRNA secretion, as well as their impact on tumor cell growth, is not yet fully understood, and requires further study [117]. The limitations of current high-throughput sequencing technologies also impede the comprehensive characterization of circRNA regulation. Large-scale, multistage clinical studies are necessary to validate circRNAs as reliable diagnostic biomarkers, addressing the challenges of small sample sizes and limited representation.

Moreover, the application of exosomal circRNAs in cancer therapy is contingent upon overcoming challenges related to their size for effective encapsulation and delivery. Pharmacokinetic studies are vital to ensure the safety and efficacy of circRNA-based therapeutics [117]. A thorough investigation of the tumor microenvironment, including diverse immune cell types and their mechanisms, is crucial for understanding T cell immune evasion in cancer. The mechanisms of resistance to immunotherapy have not yet been fully elucidated, and further experimental research is required to assess the therapeutic potential of circRNAs [3].

## 6. Perspectives and Conclusions

T cell immunity is known to be pivotal for limiting tumor growth and possibly eradicating neoplastic cells. However, T cell exhaustion poses a significant challenge in immune evasion in malignant cells. According to a previous study of human tumors, circRNAs participate in various large and complex cancer signal regulation networks. Researchers have shown that circRNAs are common in cancer cells and are vital for both tumor heterogeneity and adaptation. In this review, we systematically highlight the intricate interplay between circRNAs and T cell modulation within the tumor microenvironment. This review describes how circRNAs might impact T cell function in tumor immunity via pathways such as M6A modification and the miRNA axis, immune checkpoints, and immune activation, ultimately influencing cancer progression. Additionally, we observed a strong correlation between circRNA levels and exosomes in the tumor microenvironment, with circRNA function being subject to changes through exosome modification, suggesting a novel approach to tumor immunotherapy. The clinical application of circRNAs as targets for tumor immunotherapy and as tumor markers cannot be overlooked. Further exploration of the interaction between circRNAs and T cells may provide new insights for innovative cancer therapeutic strategies. However, our review only suggested a limited circRNA mode of action. Specific experimental validation is required to understand how circRNAs regulate T cell function in tumor immunity and immune evasion. In addition, the distinct structural advantages of circRNAs may influence other immune cells involved in tumor immunity, which requires further speculation and investigation. However, the challenges and constraints associated with utilizing circRNAs to educate T cells or augment antitumor immune responses against resilient tumors still need to be overcome. In the preanalytical and analytical phases of circRNA research, there are numerous variables to consider and challenges, biological variation, differences between starting materials, sample collection, storage conditions, RNA extraction methods, quantification and quality assessments, and profiling/measuring are a few examples. Nonetheless, investigating the impact of circRNAs may shed some light on the unknown effects of T cells and tumor cells, offering valuable insights into the molecular mechanisms underlying T cell function. This knowledge could pave the way for new strategies targeting tumor therapy, activation and modulation of T cells, and eradication of tumor resistance.

## Figures and Tables

**Table 1:** Current major immunotherapy strategies targeting T cells.

Therapy	Description	Current Limitations	References
Immune checkpoint inhibitors	Blockade of immune checkpoint receptors on T cells, such as PD-1, B7-H3, and CTLA-4, releases the brakes on antitumor T cell activity	Response only in a subset of patients; acquired resistance after the initial response	[30]
STING agonists	Activation of the STING pathway enhances dendritic cell maturation and function, leading to increased T cell-mediated antitumor immunity	No phase III trial data are available yet; mechanisms linking STING signaling and cancer immunity have not been fully elucidated	[31]
Tumor RNA vaccines	Tumor antigen-encoding mRNA or self-amplifying RNA directly translates antigens to stimulate antitumor T cell responses	High tumor heterogeneity challenges universal antigen selection; technically immature, currently in early clinical testing	[32]
CAR-T cell therapy	T cells engineered to express chimeric antigen receptors recognize and kill tumor cells expressing target antigens	Severe cytokine release syndrome; defining optimal antigens for safety and efficacy challenging	[33]
TCR-T cell therapy	T cells engineered to express tumor antigen-specific TCRs have enhanced recognition and cytotoxicity against cancers	Risk of antigen escape; severe toxicities including cytokine release syndrome and on-target off-tumor effects	[34]
CRISPR/Cas9-engineered T cells	CRISPR/Cas9 gene editing to enhance T cell proliferation, trafficking, and antitumor cytotoxicity	Low editing efficiency leads to uncertain efficacy; safety concerns due to potential genomic damage	[35]

**Table 2:** Regulation of circRNA in cancer.

circRNA	Expression	Location	Cancer	Model	Pathway	Result	Ref.
circUSP7	↑	Exosome	NSCLC	HuNSG mice	circUSP7/miR-934/SHP2	Accelerates CD8+ T cell exhaustion; Promotes resistance to anti-PD1 therapy.	[26]
circRNA-UBAP2	↑	Cytoplasm	OC	Tumor cells	circRNA-UBAP2/miR-382-5p/PRPF8	Regulates cell proliferation and apoptosis of ovarian cancer.	[80]
circRNA TCFL5	↑	Cytoplasm	EC	Tumor cells	circRNA TCFL5-FMNL2/miR-543 axis	circRNA TCFL5 encourages cell proliferation, migration, and invasion of KYSE150 and Eca109.	[81]
circCCAR1	↑	Exosome	HCC	HuNSG mice	circCCAR1/miR-127-5p/WTAP	Promotes growth and metastasis of tumor cells; promotes resistance to anti-PD1 therapy.	[25]
circRNA-002178	↑	Exosome	LUAD	Anti-Human CD279 PE Mice	circRNA-002178/miR-34a/PD-L1/ circRNA-002178/miR-28-5p/PD-1	Enhances PD-1 and PD-L1 expression.	[51]
circRNA_100395	↓	Cytoplasm	PC	Tumor cells	circRNA_100395/miR-1228	Inhibits cell proliferation; alters cell cycle distribution; reduces cell migration and invasion.	[82]
circRNA_100395	↓	Cytoplasm	BC	Tumor cells	circRNA_100395/MAPK6	Weakens proliferative and migratory abilities.	[83]
circRNA_104889	↑	Cytoplasm	LUAD	Tumor cells	circRNA_104889/ERK1/2 circRNA_104889/miR4458/CASP3	Promotes migration and invasion of lung adenocarcinoma cells.	[84]
circRNA_103993	↑	Cytoplasm	NSCLC	Tumor cells	circRNA_103993/miR-1271/ERG	Regulates proliferation and apoptosis of NSCLC cells.	[85]
circ_PTPN22	↑	Exosome	ICC	Tumor cells	_	Promotes expression level of regulatory T cells, M1 macrophages, and immune checkpoint gene.	[86]
circ_PIP5K1A	↑	exosome	NSCLC	Tumor cells	circ_PIP5K1A/miR-101/ABCC1	Regulates the progression of non-small cell lung cancer and cisplatin sensitivity.	[87]
circBART2.2	↑	exogenous circRNA	NPC	Nude mouse	circBART2.2/RIG-I/IRF3/NF-κB/PD-L1	Upregulates PD-L1 to inhibit T cell killing of NPC cells and promotes T cell apoptosis.	[76]
circRNA-HIPK3, PTK2	↑	Exosome	LC	C57BL/6 Mice	Circhipk3/PTK2/CD163/CD206/Kras	Promotes growth and metastatic potential and survival in lung tumor.	[88]
circRNA_MYLK	↑	Cytoplasm	OC	Tumor cells	circRNA_MYLK/miRNA-652	Promotes the malignant progression of OC	[89]
hsa_circRNA_100269	↓	Cytoplasm	GC	BALB/Cnude mice	hsa_circRNA_100269/PT3K/Atk	Induces the development of GC cells;suppresses cell cycle arrest and apoptosis in GC cells.	[90]
hsa_circRNA_102958	↑	Cytoplasm	OC	Tumorcells	hsa_circRNA_102958/miR-1205/SH2D3A	Promotes ovarian cancer progression.	[91]
hsa_circ_0044301	↑	cytoplasm	GC	BALB/c nude mice	hsa_circ_0044301/miRNA-188-5p/DAXX (ERK1/2)	Influences GC progression; regulates the role of the downstream target DAXX.	[92]

**Note**: BC: breast cancer; EC: esophageal cancer GC: gastric cancer; HCC: hepatocellular carcinoma; ICC: intrahepatic cholangiocarcinoma; LC: lung cancer; LUAD: lung adenocarcinoma; NPC: nasopharyngeal carcinoma; NSCLC: non-small cell lung carcinoma; OC: ovarian cancer; PC: prostate cancer. ↑/↓ represents the increase or decrease of circRNA expression.

**Table 3:** CircRNAs serve as biological markers.

circRNA	Targeting T-Cell	Cancer	Signaling Pathway	Result	Ref.
circFAM13B	CD8 T cell	BC	_	Repress immune evasion and enhance immunotherapy sensitivity.	[99]
circMET	CD8 T cell	HCC	miR-30-5p/Snail/dipeptidyl peptidase 4(DPP4)/CXCL10	Induces HCC development and immune tolerance.	[101]
circPRDM4	CD8 T cell	HCC		Promotes the expression of PD-L1 and the escape of immune cells by CD8+ T cells.	[100]
circ_14736 and circ_17720	CD8 T cell	PCa	_	Serves as a disease prognosis marker.	[102]
circRNA DYRK1A_017, circRNA FLNA_118	Regulatory T cell	GC	EMT/NFκβ-TNFα	Serves as the target of immunotherapy.	[103]
circTMTC3 and circFAM117B	CD8 T cell	Melanoma	miR-142-5p/PD-L1	Increases PD-L1 expression and reduces T cell activity leading to immune escape.	[104]
circFGFR4	CD8 T cell	TNBC	miR-1-185p/CXCR5	Serves as a biomarker for predicting sensitivity to anti-PD-1 immunotherapy and as an immunotherapeutic target.	[105]
circPVT1	_	HNSC	_	Improves and understands radiotherapy efficacy in HNCs.	[106]
circFBXW7	_	T-ALL	_	Suppresses tumors.	[107]
circCCDC134	_	NSCLC	miR-625-5p/NFAT5	Serves as the diagnostic and therapeutic target.	[108]
hsa_circ_0003763, hsa_circ_0004928, hsa_circ_0040573	_	GC	Rap1 and Ras/MAP2K1	Regulates the development of GC.	[109]
hsa_circ_0000831 …	Treg and CD4^+/^CD8^+^ T cell	NPC	_	Serves as potential biomarkers.	[110]
hsa_circ_0031594 …	_	CCRCC	_	Serves as potential biomarkers.	[111]
hsa_circ_0001583	_	BC	_	Serves as potential biomarkers.	[112]
hsa_circ_0000247	_	PDAC	_	Serves as potential biomarkers.	[113]
circADARB1	_	NKTCL	_	Serves as a biomarker to assist diagnosis.	[114]
circBCL11B	_	AML	_	Serves as biomarker and potential novel pathway dependencies	[115]
circRNA_100783	CD28-related CD8(+) T cell	_	_	Serves as potential biomarkers.	[116]

**Note:** AML: acute myelocytic leukemia; BC: breast cancer; CCRCC: clear-cell renal cell carcinoma; GC: gastric cancer; HCC: hepatocellular carcinoma; HNSC: head and neck squamous cell carcinoma; NKTCL: non-Hodgkin’s lymphoma; NPC: nasopharyngeal carcinoma; NSCLC: non-small cell lung carcinoma; PCa: prostate cancer; PDAC: pancreatic ductal adenocarcinoma; T-ALL: T cell acute lymphoblastic leukemia; TNBC: triple-negative breast cancer.

## Data Availability

No new data were created or analyzed in this study.
